# Reducing Antimicrobial Resistance in Poultry Carcasses Extends Beyond Farm-Level Interventions

**DOI:** 10.3390/foods15081440

**Published:** 2026-04-21

**Authors:** Valentina Indio, Yitagele Terefe Mekonnen, Chiara Oliveri, Sofia Rubboli, Marco Candela, Alessandro Seguino, Andrea Serraino, Alessandra De Cesare

**Affiliations:** 1 Department of Veterinary Medical Sciences, University of Bologna, Ozzano dell’Emilia, 40064 Bologna, Italyalessandra.decesare@unibo.it (A.D.C.); 2 Department of Pharmacy and Biotechnology, University of Bologna, Ozzano dell’Emilia, 40064 Bologna, Italy

**Keywords:** antibiotic-free flocks, broiler caeca, broiler carcasses, resistome, shotgun sequencing

## Abstract

The aim of this study was to assess how raising chickens without the use of antimicrobials affects the microbiome of poultry carcasses. A total of 151 caeca and neck skin samples from chickens raised without antimicrobials were collected in the same slaughterhouse and submitted to shotgun metagenomic sequencing. Caeca were dominated by Bacillota and Bacteroidota, while carcasses by Pseudomonadota. The caeca microbiome was enriched in genes related to a proliferating and metabolically active microbial community. Carcass-associated microbiomes were enriched in functional genes linked to adaptation to nutritionally limited and oxidative environments. A significantly higher cumulative antimicrobial resistance gene abundance was detected in carcasses compared to caeca. Specifically, carcasses exhibited approximately 1.5 times more AMR genes, reflecting an increase of nearly 49%. While caeca showed enrichment of resistance determinants associated with Gram-positive anaerobic gut commensals, carcasses were characterized by a predominance of multidrug efflux systems and clinically relevant β-lactam resistance genes, commonly associated with environmental and opportunistic Gram-negative bacteria. In carcasses, carbapenem-associated genes, such as OXA-58-like and CphA, were detected. However, these genes have not been associated with carbapenemase-producing Enterobacterales. Overall, the findings of this study indicate that reducing antimicrobial resistance in food animal production systems extends beyond farm-level intervention. At present, the benefits of the interventions aimed at reducing antimicrobial resistance at farm level seem to be compromised during the post-harvest stages.

## 1. Introduction

In the European Union (EU), antimicrobial resistance (AMR) is estimated to cause more than 35,000 human deaths each year [1]. Moreover, the annual economic burden of AMR is estimated at €1.5 billion. AMR encompasses human, animal and environmental health, thus representing a One Health issue. In 2017, the EU adopted a One Health Action Plan Against AMR. However, the established AMR reduction targets have not yet been achieved. There are multiple reasons why the expected targets have not been met. One key reason is that AMR is a multi-faceted cross-border health threat that cannot be tackled without a systematic approach. Tackling AMR requires a high level of collaboration across sectors and between countries, including at the global level. One sector in which AMR has not yet been addressed systematically is food animal production systems. In this sector, all the efforts to fight AMR have primarily focused on the farm level [2,3,4].

According to a recent market report from the European Commission, poultry meat production in the EU reached approximately 14.1 million tonnes in 2024, representing a notable 6.0% increase compared to 2023. Poultry is one of the animal production systems most prone to being raised without the use of antimicrobials because the chicken life cycle is very short (i.e., 35–42 days). The strategies to raise chickens without using antimicrobials include a vaccination program, implementation of robust biosecurity measures, training of farmers, and use of microbiomes to fight pathogen colonization in the gastrointestinal tract [5]. Besides the chicken gastrointestinal tract, litter can also be kept free of pathogens by using probiotics [6,7]. Tang et al. demonstrated that reducing the level of antibiotic use in animal production can be effective in fighting antibiotic resistance in both animals and humans [8]. To limit the use of antibiotics in animal rearing, Regulation (EC) No. 1831/2003 [9] banned the market and use of antibiotics as growth promoters in feed in the European Union in January 2006. More recently, Regulation EU 2019/6 [10] laid down rules for the placing on the market, manufacturing, import, export, supply, distribution, pharmacovigilance, control and use of veterinary medicinal products. Moreover, Regulation EU 2019/4 [11] targeted the manufacture, placing on the market and use of medicated feed.

In 2021, the European Food Safety Authority (EFSA) published a scientific opinion on the role of the environment in the emergence and spread of AMR through the food chain [12]. The document highlights that the slaughter line can introduce and spread antimicrobial-resistant bacteria during defeathering and evisceration through spillage of gastrointestinal content [13,14,15]. Contamination can also originate from water used in the slaughtering process [16], from workers, through their hands and from air and aerosol [14]. At present, studies on poultry carcass resistome are lacking. The few studies available concern pig slaughterhouses [17,18]. In 2022 EFSA published a second scientific opinion on the transmission of AMR bacteria and genes between animals, including chickens, during transport [19]. It has been documented that such transmission is due to a number of factors, including fecal shedding during transport, poor hygiene of vehicles and lairage areas, and transport duration.

In 2022, we conducted a pilot study to quantify differences in antimicrobial resistance load between caeca and corresponding carcasses at slaughtering. The primary research question was whether raising chickens without the use of antimicrobials affects the microbiome of poultry meat. To address this question, we compared the microbiomes of caeca, collected during evisceration, with those of the corresponding carcasses sampled at the end of the chilling tunnel. Caeca and carcasses originated from two flocks of chickens reared either with or without antimicrobials, respectively [20]. The results showed that caeca from chickens raised in the antibiotic-free flock had a significantly lower AMR load than those from the conventionally reared flock. However, the AMR load detected on carcasses from the two flocks was comparable, showing that post-harvest steps negate the positive impact of antibiotic-free rearing conditions on the carcass microbiome. In the present study, we expanded our investigation, considering only antibiotic-free flocks to confirm the increase in AMR genes between caeca and carcasses. A total of 151 samples were tested, including four antibiotic-free poultry flocks sampled before and after the pandemic period. The tested poultry flocks were reared in the same poultry houses during the different sampling periods and were slaughtered in the same facility.

## 2. Materials and Methods

### 2.1. Experimental Design

A total of 152 caeca and neck skin samples were collected in the same slaughterhouse. The samples belonged to 76 broilers from four antibiotic-free flocks, reared in two poultry houses, labeled as CM and ZR. A total of 15 broilers from each of the two poultry houses were slaughtered between 12 and 21 November 2019. A total of 23 broilers from each of the two poultry houses were slaughtered between 1 and 10 March 2023. The sample characteristics are summarized in Table 1.

At the slaughterhouse, from each broiler, both the gastrointestinal tract and the whole carcass were sampled. Each gastrointestinal tract was collected at the evisceration machine, placed in a sterile plastic bag and transported to the laboratory in refrigeration conditions. Each carcass was sampled at the end of the refrigeration tunnel, placed in a sterile plastic bag and transported to the laboratory under refrigeration conditions. The sampling was organized in order to collect the gastrointestinal tract and carcass of the same animal, labeling the hook of the carcass from which the gastrointestinal tract was sampled.

In the laboratory, the caeca were separated from the gastrointestinal tract. The caeca content was squeezed into 2 mL plastic tubes, flash-frozen in liquid nitrogen and stored at −80 °C until DNA extraction. From each carcass, approximately 10 g of neck skin was dissected, diluted 1:10 with sterile physiological solution (NaCl 0.90%), homogenized in the stomacher MAYO Homogenius HG400V (Mayo International srl, Novate Milanese, Italy)at normal speed for 1 min and centrifuged at 4 °C for 20 min at 6800 rpm. The supernatant was discarded, while the pellet was stored at −80 °C until DNA extraction.

### 2.2. DNA Extraction and Sequencing

Total DNA was extracted from the caeca contents using the QIAmp^®^ DNA Stool Mini Kit (Qiagen, Milan, Italy), as previously described [21]. Moreover, DNA was extracted from neck skin pellet using the PowerFood^®^ Microbial DNA Isolation Kit (Qiagen, Mo Bio Laboratories, Hilden, Germany), as previously described [22]. The DNA quality and quantity, achieved from the DNA extraction, were measured using the BioSpectrometer^®^ (Eppendorf, Milan, Italy). The extracted DNAs were fragmented and tagged with sequencing indexes and adapters using the Nextera XT DNA Library Preparation Kit (Illumina, Inc., San Diego, CA, USA). Shotgun metagenomic sequencing was performed using the NextSeq500 (Illumina) at 2 × 150 bp in paired-end mode.

### 2.3. Bioinformatic and Biostatistics Analysis

Raw sequencing reads were processed using bcl2fastq to perform adapter trimming and quality filtering, retaining only high-quality reads for downstream analyses. Host-derived sequences were removed by aligning the filtered reads to the *Gallus gallus* reference genome (Galgal6) with Bowtie2 and discarding all reads mapping to the host. Taxonomic classification of the remaining microbial reads was carried out with Kaiju using the nr_euk protein reference database to assign taxa based on protein-level homology [23]. The taxonomic profiles were normalized to relative abundances, which inherently accounts for differences in sequencing depth across samples.

Diversity indices were calculated using the Shannon index to assess the microbial richness, and the Bray–Curtis method was used to compute the beta diversity. Both indices were calculated using the R package *vegan* (respectively *diversity* and *vegdist*) starting from the genus-level relative abundances. The beta diversity distance matrix was used to compute the Principal Coordinates Analysis (R package *stats*, function *cmdscale*) to reduce the data to three dimensions, which were visualized in a 3D scatter plot (R package *rgl*).

To perform the functional characterization of the microbial communities, paired-end sequencing reads were concatenated for each sample and analyzed with HUMAnN3 (v3.9) using the UniRef90 protein database as reference [24]. The resulting pathway abundances were then normalized to counts per million (CPM) for consistent comparison across samples.

Antimicrobial resistance (AMR) genes were detected using the Resistance Gene Identifier (RGI) with the CARD as the reference [25]. Only alignments at the allele level showing more than 95% identity to the CARD reference and covering at least 50 base pairs were retained and normalized, as previously described [20], using the Fragments Per Kilobase per Million mapped reads (FPKM) approach. FPKM accounts for both gene length and sequencing depth, enabling more accurate comparisons between AMR gene abundance across samples with different library sizes. The aggregated results were then calculated at the AMR gene family and drug class levels by summing the allele-level counts.

Statistical analyses were performed using the Wilcoxon rank-sum test for pairwise comparisons between groups. Differences in beta diversity were evaluated using permutational multivariate analysis of variance (Adonis). For all analyses, results were considered statistically significant at *p* < 0.05.

Data visualization was performed under the R environment with the *ggplot2* package to generate graphical representations of taxonomic, functional, and AMR profiles.

## 3. Results

A total of 151 metagenomes were collected in this study: 15 from caeca and 15 from the neck skin of broilers reared in the poultry house CM sampled in 2019; 15 from caeca and 15 from neck skin of broilers reared in the poultry house ZR sampled in 2019; 22 from caeca and 23 from neck skin of broilers reared in the poultry house CM sampled in 2023; 23 from caeca and 23 from neck skin of broilers reared in the poultry house ZR sampled in 2023. One caeca sample from the poultry house CM sampled in 2023 did not result in good DNA quality and was discarded. The other 151 samples were sequenced, yielding an average of 5.25 Gb per sample.

### 3.1. Taxonomic Profile

**Figure 1:**
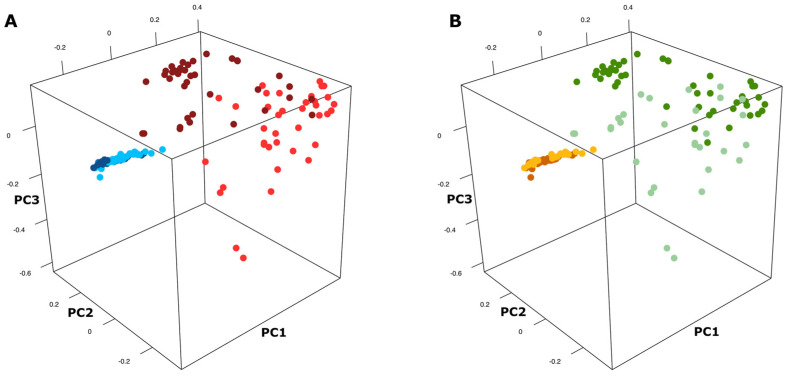
Three-dimensional projection of PCoA derived from Bray–Curtis beta diversity calculation for the different poultry houses (**A**) and sampling time (**B**). Panel (**A**)—Deep blue: caeca of broilers reared in the poultry house CM; Light blue: caeca of broilers reared in the poultry house ZR; Deep red: carcasses of broilers reared in the poultry house CM; Light red: carcasses of broilers reared in the poultry house ZR. Panel (**B**)—Light orange: caeca sampled at T1; Deep orange: caeca sampled at T2; Light green: carcasses sampled at T1; Deep green: carcasses sampled at T2.

The genus-level β-diversity analysis, based on the Bray–Curtis dissimilarity metric, revealed pronounced differences in microbial community structure among the tested samples. A significant taxonomic compositional divergence was observed between caeca and carcass samples (adonis adj. *p*-value < 0.001), with a higher homogeneity among caeca (Figure 1A). The beta diversity divergence was significantly pronounced in carcasses originating from the two poultry houses (adonis adj. *p*-value = 0.003), while it was not observed in caeca (adonis adj. *p*-value = 0.690) (Figure 1A). Moreover, sampling time emerged as a factor deeply associated with measurable changes in microbial composition in both caeca and carcasses (respectively adonis adj. *p*-value < 0.001 and adj. *p*-value = 0.009) (Figure 1B). These results indicate that sample type (i.e., caeca vs. carcasses) and sampling period (i.e., T1 = 2019 vs. T2 = 2023) were the primary drivers of community variation in this dataset, while the poultry house had an impact on carcass microbiome but not on caeca microbiome shifts.

**Figure 2:**
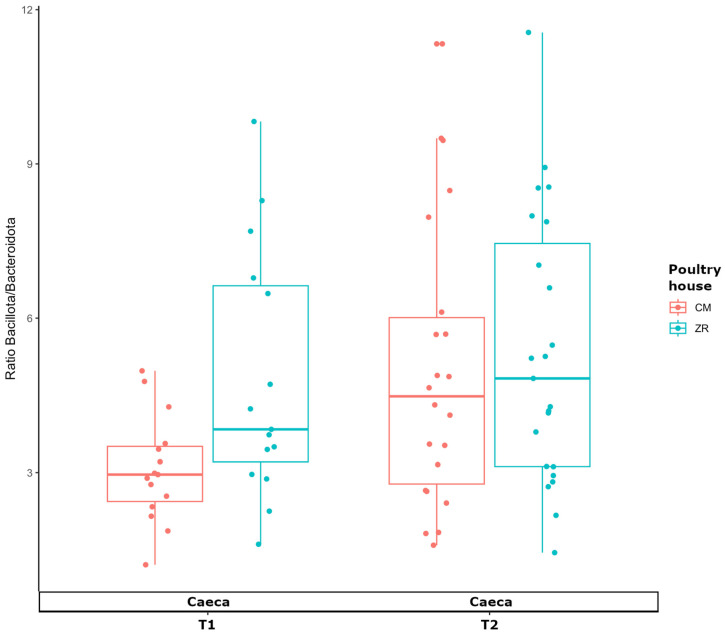
Box plot showing the ratio between Bacillota and Bacteroides (F/B) in the microbiome of caeca of broilers reared in the poultry houses CM and ZR at T1 and T2.

The evaluation of taxonomic composition at the phylum level revealed that the caeca were dominated by Bacillota (71.7%) and Bacteroidota (19.0%), together accounting for over 90% of the total community. In contrast, the carcass microbiome showed a distinct composition, with Pseudomonadota representing the predominant phylum (51.7%), followed by Bacillota (20.9%). Other phyla, including Chlamydiota (7.4%) and Actinomycetota (7.3%), were detected at moderate relative abundances, whereas Bacteroidota represented only a minor fraction (2.5%). Additionally, carcass samples exhibited a higher proportion of unclassified taxa (9.3%) compared with cecal samples (2.4%), indicating greater taxonomic heterogeneity. Interestingly, the ratio between Bacillota (formerly Firmicutes) and Bacteroidota in the caeca was significantly higher at T2 than at T1 in samples collected from the CM poultry house (*p*-value = 0.034), whereas no significant difference was observed in ZR (*p*-value = 0.531). At T1, ZR exhibited a significantly higher F/B ratio compared with the CM poultry house (*p*-value = 0.037); however, this difference was no longer evident at T2 (*p*-value = 0.642) (Figure 2).

**Figure 3:**
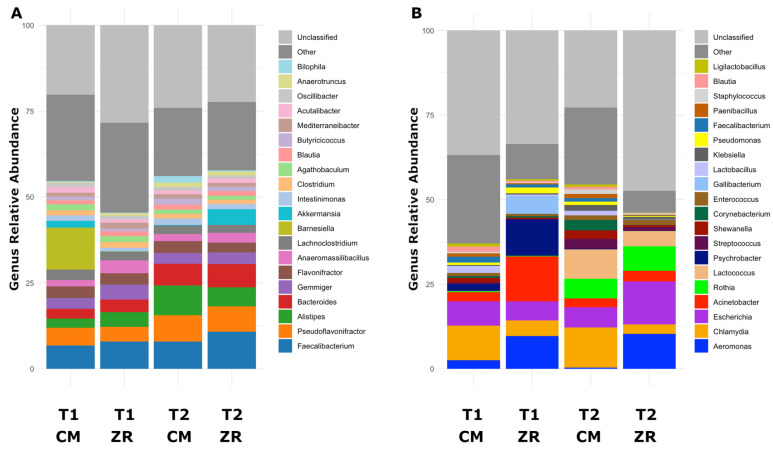
Mean relative abundance of the top 20 genera quantified in caeca (**A**) and carcasses (**B**) from the two poultry houses (i.e., CM and ZR) sampled at both sampling times (T1, 2019; T2, 2023).

Considering the top 20 most abundant genera identified in caeca and carcasses, different compositional profiles were highlighted (Figure 3). *Faecalibacterium*, followed by *Pseudoflavonifractor*, *Alistipses* and *Bacteroides* were the most abundant genera in caeca, showing a homogeneous taxonomic composition along time points and poultry houses (Figure 3A). In contrast, in carcasses, the taxonomic composition was more divergent between groups (Figure 3B). In poultry house ZR, a higher abundance of *Aeromonas* (*p* = 3.51 × 10^−13^; FDR = 2.03 × 10^−10^) and a lower abundance of *Chlamydia* (*p* = 2.36 × 10^−10^; FDR = 1.56 × 10^−8^) were quantified compared to CM. Moreover, on carcasses originating from both poultry houses, at T1 the relative abundance of *Psychrobacter* was significantly higher compared to T2 (*p* = 2.47 × 10^−18^; FDR = 3.11 × 10^−15^), while a high abundance of *Rothia* was quantified at T2. This genus was not detected at T1 (*p* = 5.81 × 10^−16^; FDR = 3.66 × 10^−13^) (Figure 3B).

**Figure 4:**
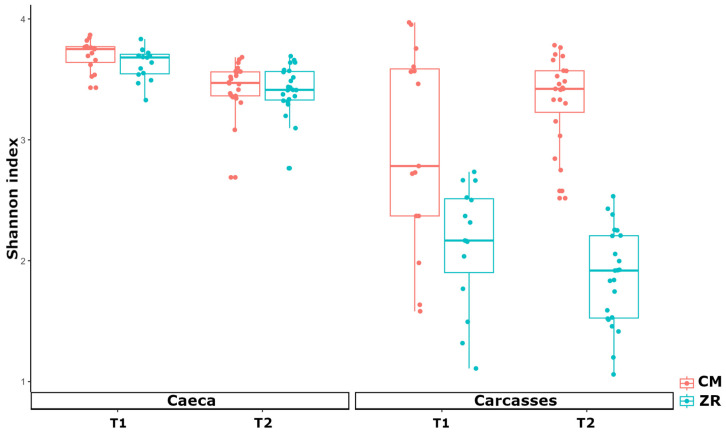
Box plots of the alpha diversity indexes calculated at the genus level by the Shannon index for caeca and carcass samples collected from the tested poultry houses (i.e., CM and ZR) at both sampling times (T1, 2019; T2, 2023).

In terms of alpha diversity, reflecting both the richness and evenness of bacterial genera, the Shannon index was significantly higher in cecal samples than in carcass samples (*p* < 0.001). Cecal microbiome exhibited consistently high alpha diversity with comparable values between poultry houses CM and ZR; however, a significant reduction from T1 to T2 was observed in both poultry houses (*p* < 0.001 in CM and *p* = 0.001 in ZR). In contrast, carcass samples showed lower alpha diversity, with carcasses from poultry house CM displaying significantly higher values than those from poultry house ZR at both time points (*p* = 0.006 at T1 and *p* < 0.001 at T2) (Figure 4).

### 3.2. Functional Profile

**Figure 5:**
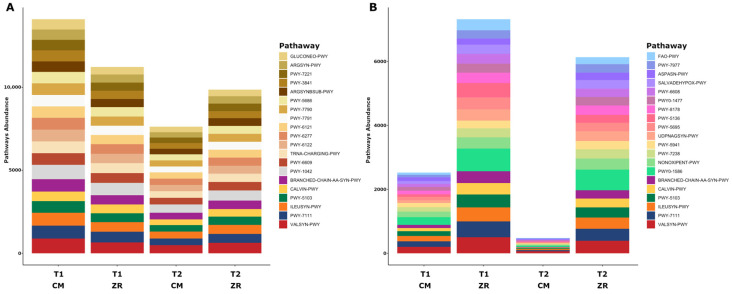
MetaCyc pathway abundance evaluation in the microbiomes of caeca and carcasses collected in the two poultry houses (i.e., CM and ZR) at both sampling times (T1, 2019; T2, 2023). Panel (**A**) Top 20 most abundant pathways in caeca: VALSYN-PWY: L-valine biosynthesis; PWY-7111: pyruvate fermentation to isobutanol (engineered); ILEUSYN-PWY: L-isoleucine biosynthesis I (from threonine); PWY-5103: L-isoleucine biosynthesis III; CALVIN-PWY: Calvin–Benson–Bassham cycle; BRANCHED-CHAIN-AA-SYN-PWY: superpathway of branched-chain amino acid biosynthesis; PWY-1042: glycolysis IV; PWY-6609: adenine and adenosine salvage III; TRNA-CHARGING-PWY: tRNA charging; PWY-6122: 5-aminoimidazole ribonucleotide biosynthesis II; PWY-6277: superpathway of 5-aminoimidazole ribonucleotide biosynthesis; PWY-6121: 5-aminoimidazole ribonucleotide biosynthesis I; PWY-7791: UMP biosynthesis III; PWY-7790: UMP biosynthesis II; PWY-5686: UMP biosynthesis I; ARGSYNBSUB-PWY: L-arginine biosynthesis II (acetyl cycle); PWY-3841: folate transformations II (plants); PWY-7221: guanosine ribonucleotides de novo biosynthesis; ARGSYN-PWY: L-arginine biosynthesis I (via L-ornithine); GLUCONEO-PWY: gluconeogenesis I. Panel (**B**) Top 20 most abundant pathways in carcasses: VALSYN-PWY: L-valine biosynthesis; PWY-7111: pyruvate fermentation to isobutanol (engineered); ILEUSYN-PWY: L-isoleucine biosynthesis I (from threonine); PWY-5103: L-isoleucine biosynthesis III; CALVIN-PWY: Calvin–Benson–Bassham cycle; BRANCHED-CHAIN-AA-SYN-PWY: superpathway of branched-chain amino acid biosynthesis; PWY0-1586: peptidoglycan maturation (meso-diaminopimelate containing); NONOXIPENT-PWY: pentose phosphate pathway (non-oxidative branch) I; PWY-7238: sucrose biosynthesis II; PWY-5941: glycogen degradation II; UDPNAGSYN-PWY: UDP-N-acetyl-D-glucosamine biosynthesis I; PWY-5695: inosine 5’-phosphate degradation; PWY-5136: fatty acid & beta;-oxidation II (plant peroxisome); PWY-8178: pentose phosphate pathway (non-oxidative branch) II; PWY0-1477: ethanolamine utilization; PWY-6608: guanosine nucleotides degradation III; SALVADEHYPOX-PWY: adenosine nucleotides degradation II; ASPASN-PWY: superpathway of L-aspartate and L-asparagine biosynthesis; PWY-7977: L-methionine biosynthesis IV; FAO-PWY: fatty acid & beta-oxidation I (generic).

Figure 5 displays the top 20 most represented metabolic pathways identified in the microbial community of cecal contents and carcasses tested in this study. This descriptive analysis aims to highlight the most abundant pathways in each matrix. Among the 20 most abundant pathways identified in the microbiome of caecal contents and carcasses, six were shared between the two matrices.

The shared pathways included L-valine biosynthesis (VALSYN-PWY), pyruvate fermentation to isobutanol (PWY-7111), L-isoleucine biosynthesis I from threonine (ILEUSYN-PWY), L-isoleucine biosynthesis III (PWY-5103), the Calvin–Benson–Bassham cycle (CALVIN-PWY), and the superpathway of branched-chain amino acid biosynthesis (BRANCHED-CHAIN-AA-SYN-PWY) (Figure 5).

In addition to these pathways, predominantly associated with protein synthesis, the metabolic functions most prominently detected in the caeca were related to carbon metabolism with glycolysis IV (PWY-1042) and gluconeogenesis I (GLUCONEO-PWY) supporting energy production and metabolic flexibility. In parallel, multiple pathways involved in nucleic acids biosynthesis were detected, including uridine monophosphate (UMP) biosynthesis I, II and III (PWY-5686, PWY-7790, PWY-7791), guanosine ribonucleotides de novo biosynthesis (PWY-7221), and 5-aminoimidazole ribonucleotide biosynthesis I–III (PWY-6121, PWY-6122, PWY-6277) together with folate-mediated one-carbon metabolism (PWY-3841), reflecting an increased demand for nucleotides. The enrichment of tRNA charging (TRNA-CHARGING-PWY) and L-arginine biosynthesis pathways (ARGSYN-PWY, ARGSYNBSUB-PWY) further supports an elevated rate of protein synthesis. Overall, this metabolic profile is consistent with the presence of a microbial community showing active bacterial proliferation and high metabolic activity (Figure 5A).

In contrast, the metabolic pathways identified in the carcasses were related to glycogen degradation I and II (FAO-PWY; PWY-5941), sucrose biosynthesis II (PWY-7238), fatty acid β-oxidation (PWY-5136), and the pentose phosphate pathway (PWY-8178). These pathways highlight microbial activity under nutritionally limited conditions and focus on cellular maintenance rather than proliferation (Figure 5B).

### 3.3. Resistome Profile

**Figure 6:**
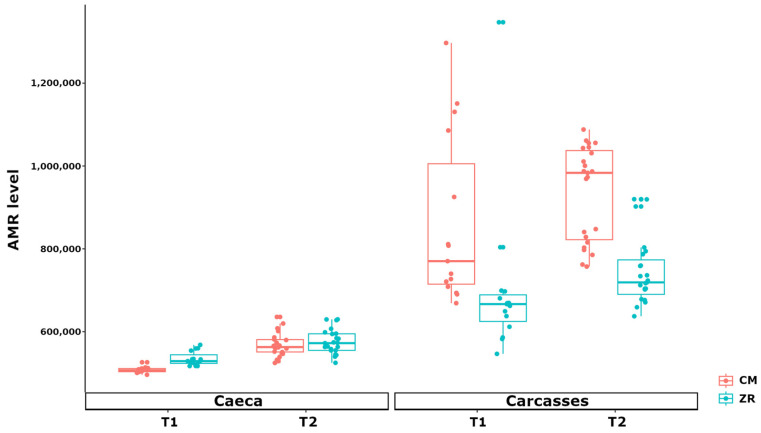
Box plots showing the total AMR level (FPKM) per type of sample (i.e., caeca and carcasses), stratified by poultry house (i.e., CM vs. ZR) and sampling time (T1 vs. T2).

The resistome analysis performed with the RGI pipeline displayed remarkable differences in antibiotic resistance ontologies (ARO), defining a different AMR profile in caeca compared to carcasses. The total abundance of AMR genes differed between sample matrices, poultry houses, and sampling time points (Figure 6).

Overall, carcass samples showed a significantly higher cumulative AMR load than caeca across poultry houses and time points (*p* < 0.001). In caeca, AMR levels were comparable between poultry houses, with slightly higher values observed in ZR compared to CM only at T1 (*p* < 0.001). In addition, an increase in cumulative AMR gene abundance was detected at T2 in both poultry houses (*p* < 0.001 in CM and *p* < 0.001 in ZR). In contrast, carcasses showed both a higher abundance of AMR genes and increased variability. At both T1 and T2, carcasses from the poultry house ZR showed lower AMR abundance compared to carcasses from CM (*p* < 0.001 at T1 and *p* < 0.001 at T2). Moreover, a significantly higher AMR level was detected on carcasses from the poultry house ZR in the comparison between T2 and T1, while no difference was observed in the comparison between T1 and T2 for carcasses from CM. The results displayed in Figure 6 show a clear distinction in AMR gene load between caeca and carcass samples and also between poultry houses and time points.

Table 2 shows the top 10 most represented AMR gene families in carcasses (negative log2FC) and caeca (positive lof2FC) together with the putative drug class and resistance mechanisms.

Carcasses were characterized by a higher representation of genes associated with intrinsic and multidrug resistance mechanisms, including resistance–nodulation–cell division (RND), major facilitator superfamily (MFS), and small multidrug resistance (SMR) efflux pumps. In contrast, caeca showed a marked overrepresentation of genes associated with specific drug resistance, such as the 23S rRNA mutations associated with pleuromutilin, the streptothricin acetyltransferase (nucleoside), the glycopeptide resistance gene cluster and several aminoglycoside-modifying enzymes (APH, ANT, rpsL).

**Figure 7:**
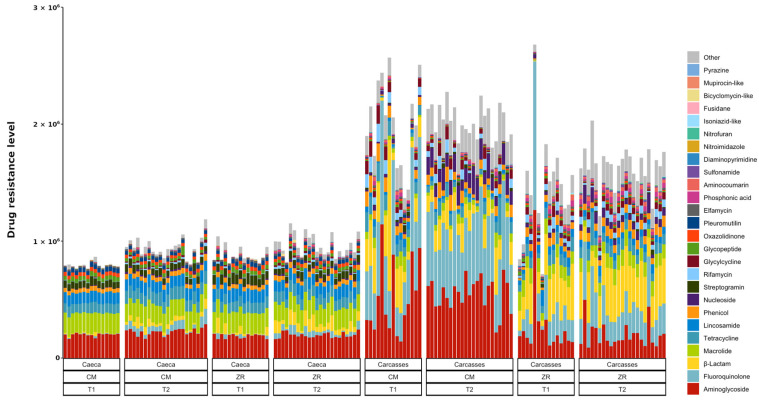
Stacked bar plot of antimicrobial resistance abundance (FPKM) per drug class (colors) per sample.

Antibiotic resistance genes associated with β-lactam resistance were detected in both caeca and carcasses but were overrepresented in carcasses. In caeca and carcasses, distinct gene sets were identified: carcasses were characterized by a significantly higher abundance of OXA-12-like, OXA-58-like, AmpC, and CphA genes, while OXA-184-like and CepA were predominantly associated with caeca. It is important to highlight that CphA and OXA-58-like genes confer resistance to carbapenems. Since carbapenems are used for treatment of severe or multidrug-resistant bacterial human infections when other antibiotics are no longer effective, the identification of genes coding resistance to carbapenem in carcasses is of particular concern. Figure 7 displays the stacked bar plots showing the summative abundance of AMR genes, expressed as normalized abundance, that contributed to the resistance to the specific drug classes.

The RGI analysis revealed significant differences in the abundance of antibiotic resistance genes (ARGs) between caeca and carcasses (Figure 7 and Table 3). The comparative analysis showed several resistance classes that were significantly more abundant in carcasses compared to caeca (negative log2 fold change), including fluoroquinolone, glycylcycline, β-lactam, phosphonic acid, aminocoumarin, nucleoside, and rifamycin resistance genes, all showing highly significant *p*-values (*p* < 0.001). Among these, fluoroquinolone and β-lactam resistance genes displayed the strongest enrichment in carcasses, with log2 fold changes of −4.24 and −3.01, respectively. Conversely, several drug resistances were significantly enriched in caeca (positive log2 fold change values). Notably, resistance genes associated with pleuromutilins (log2FC = 3.65), streptogramins (log2FC = 3.50), mupirocin-like antibiotics (log2FC = 3.97), and bicyclomycin-like antibiotics (log2FC = 4.06) were strongly enriched in caeca contents. Additional significant enrichments were observed for macrolides, lincosamides, glycopeptides, oxazolidinones, and elfamycins (all *p* < 0.001). Phenicol and aminoglycoside resistance genes were significantly different between caeca and carcasses but exhibited small effect sizes (|log2FC| < 1). In contrast, nitrofuran, isoniazid-like, and diaminopyrimidine resistance classes did not display statistically significant differences between caeca and carcasses. Overall, these results indicate a distinct distribution of antibiotic resistance gene profiles between caeca and carcasses, highlighting sample-type–specific enrichment patterns for multiple clinically relevant antibiotic classes.

## 4. Discussion

In the present study, we expanded a previous investigation to assess to what extent the microbiome and, specifically, the resistome of broiler caeca differ from those of corresponding carcasses, testing four antibiotic-free poultry flocks, sampled before and after the COVID-19 pandemic. The tested poultry flocks were reared in the same poultry houses during the different sampling periods and were slaughtered in the same facility.

The marked β-diversity separation between caeca and carcass microbiomes confirmed that the slaughtering process profoundly affects the carcass microbiome. Caeca were dominated by Bacillota and Bacteroidota, a composition consistent with the well-established structure of the chicken gut microbiome, typically enriched in strict anaerobes [26]. The high alpha diversity and compositional homogeneity observed in the caeca reflect the ecological stability of the chicken gut, where moderated fluctuation in the F/B ratio was observed. This ratio has been proposed as an indicator of the gastrointestinal function efficiency and microbial eubiosis [27]. However, numerous studies, especially in mammalian models, have pointed out that the F/B ratio alone is a limited biomarker, as similar compositional profiles can be observed across a wide range of host health conditions. Therefore, relative changes in Firmicutes or Bacteroidetes abundances must be interpreted in the context of other microbiota features and host physiology [28].

Carcasses were characterized by a predominance of Pseudomonadota, suggesting a strong environmental impact during the transport and slaughtering steps that favor aerotolerant and psychrotrophic taxa [29]. The enrichment of genera such as *Psychrobacter*, *Aeromonas*, and *Rothia* confirms that the carcass microbiome is shaped not only by possible cross-contaminations with the intestinal contents during evisceration but primarily by environmental contamination in the processing line, coming from water, surfaces, aerosols, and even workers. This finding aligns with previous evidence indicating that the transport and slaughtering processes may act as ecological mixing chambers, facilitating cross-contamination events and microbial redistribution [30].

The temporal shift observed between T1 (pre–COVID-19) and T2 (post–COVID-19), in both caeca and carcass microbiomes, suggests that changes in farm management, biosecurity reinforcement, alterations in workforce organization, sanitation practices, or supply chain disruptions, during and after the pandemic, could have indirectly shaped microbial ecology at both poultry house and processing levels [31].

The functional profiling revealed relevant metabolic differences between the tested matrices. The caeca microbiome was enriched in pathways related to central carbon metabolism and amino and nucleotide acid biosynthesis, indicating an actively proliferating and metabolically robust microbial community. These functions are coherent with the nutrient-rich, anaerobic intestinal environment, where microbial fermentation supports rapid biomass turnover. Conversely, carcass-associated microbiomes were enriched in function genes associated with pathways involved in glycogen degradation, fatty acid β-oxidation, pentose phosphate pathway activity, and alternative energy metabolism. Such metabolic signatures are consistent with bacteria adapting to nutritionally limited and oxidative environments, prioritizing maintenance and stress resistance over rapid growth. This functional shift reinforces the ecological interpretation that carcasses represent a selective environment favoring stress-tolerant and metabolically flexible taxa [32].

The functional profiles identified in the caeca contents and carcasses are consistent with their taxonomic compositions. In the caeca, the high abundance of Bacillota and Bacteroidota, including genera such as *Faecalibacterium*, *Bacteroides* and *Alistipes*, is associated with active carbohydrate fermentation and biosynthetic metabolism. The listed phyla and genera are well known for their ability to degrade complex substrates and sustain high rates of bacterial growth [33,34]. Therefore, their presence aligns with the observed enrichment of pathways involved in amino acid biosynthesis, nucleotide production, glycolysis, and gluconeogenesis observed in the caeca. In contrast, the phylum Pseudomonadota and the genera *Pseudomonas*, *Aeromonas*, *Acinetobacter* and *Psychrobacter* were primarily identified on carcasses. These genera are commonly associated with environmental persistence and metabolic versatility under nutrient-limited conditions [35,36]. Therefore, the carcass taxonomic composition is consistent with the functional enrichment of pathways related to energy extraction and cellular maintenance, including fatty acid β-oxidation, glycogen degradation, and the pentose phosphate pathway. Overall, these findings indicate that the functional differences detected between caeca and carcass microbiomes are consistent with their different taxonomic structures, reflecting a shift from a highly proliferating microbial community in the caeca to a more stress-adapted microbiome on carcasses.

One of the most relevant findings of this study is the significantly higher cumulative AMR gene abundance detected in carcasses compared to caeca. This observation indicates that post-harvest stages can override the beneficial impact of antibiotic-free rearing conditions on the carcass resistome, confirming and extending our previous findings [20]. Focusing on carcass samples, a significantly lower AMR load was observed on carcasses originating from poultry house ZR compared to CM at both sampling time points. To explain this observation, the first hypothesis is that, in both sampling times, flocks from poultry house CM were slaughtered in the afternoon, whereas those from poultry house ZR were processed in the morning. As a result, the latter may have been exposed to lower levels of cross-contamination due to cleaner environmental conditions at the beginning of the working day. Notably, the slaughterhouse was the same for both broiler flocks at both sampling times, and slaughtering occurred approximately four days apart for the two flocks. The second hypothesis might be related to the distance between the poultry house and the slaughtering facility. The poultry house ZR was much closer than CM to the slaughterhouse (approximately 43 vs. 99 km), and a shorter transport time might have limited the fecal shedding of microorganisms, including AMR microorganisms, between animals in the truck [19]. The contamination occurring during transport may not have been fully eliminated during the slaughtering process, thereby contributing to the observed differences between AMR in carcasses originating in the different poultry houses.

While caeca showed enrichment of resistance determinants typically associated with Gram-positive anaerobic gut commensals (e.g., pleuromutilin, streptogramin, glycopeptide, and certain aminoglycoside resistance genes), carcasses were characterized by a predominance of multidrug efflux systems (RND, MFS, SMR) and clinically relevant β-lactam resistance genes. Efflux pump overrepresentation suggests selection for intrinsic and broad-spectrum resistance mechanisms, which are commonly associated with environmental and opportunistic Gram-negative bacteria [37]. The presence of such genetic determinants indicated several antimicrobial classes that were significantly more abundant in carcasses than in caecal samples, namely fluoroquinolones, β-lactams, glycylcyclines, phosphonic acids, nucleosides and aminocoumarins. The strong enrichment of fluoroquinolone and β-lactam resistance genes in carcasses further supports the hypothesis that processing environments select for bacteria exposed to antimicrobial pressures, possibly originating from water systems or persistent biofilms. In contrast, antimicrobial classes enriched in caeca appeared to be more reflective of the intrinsic resistome of the commensal gut microbiome.

Fluoroquinolones are broad-spectrum bactericidal antibiotics classified by the World Health Organization as the highest priority critically important antimicrobials for human medicine due to their essential role in the treatment of severe bacterial infections and the limited availability of therapeutic alternatives [38]. The higher abundance of fluoroquinolone resistance genes detected on carcasses is consistent with the most recent EFSA and ECDC EU Summary Report on AMR in zoonotic and indicator bacteria from humans, animals and food in 2022–2023. In the document, the frequent detection of fluoroquinolone-resistant bacteria in poultry meat is reported, highlighting the potential contribution of slaughter and processing environments to the dissemination of resistance [39].

In the present study, we detected the presence of carbapenem-associated genes, such as OXA-58-like and CphA, in carcasses. However, the isolation of carbapenem-resistant microorganisms was not performed. Carbapenems are considered last-resort antimicrobials in human medicine and are included in the WHO List of Medically Important Antimicrobials within the group “authorized for use in humans only”, which comprises agents considered of highest priority due to their critical role in the treatment of multidrug-resistant infections [38]. The detection of genes conferring resistance to carbapenem in poultry carcasses underscores the importance of the slaughterhouse settings for the dissemination of AMR. In a recent opinion, EFSA drew attention to the bacteria primarily involved in carbapenem resistance, namely carbapenemase-producing Enterobacterales (CPE), emphasizing the need to expand monitoring to food and environmental matrices not currently included in official surveillance programs. These include fish and aquaculture products, foods of non-animal origin, food production environments, animal feed and emerging food categories, such as edible insects. EFSA also recommends extending surveillance to a broader range of bacterial species, beyond traditional indicators, with particular attention to Enterobacteriales commonly associated with human infections, while also considering environmental and aquatic species [40].

In our study, due to the high sensitivity of the shotgun metagenomic approach and the complexity of the chicken microbiome, it was possible to detect two carbapenem-hydrolyzing enzymes, CphA and OXA-58, associated with specific bacterial groups. These two genes are discussed in the EFSA Opinion [40], although they are not classified among those of the highest clinical relevance. In fact, they are not typical of Enterobacterales, are usually detected at low abundance, may belong to environmental or opportunistic bacteria, and do not automatically imply clinically expressed resistance. Specifically, CphA belongs to Ambler class B, as it is a metallo-β-lactamase. Although it is a true carbapenemase, capable of directly hydrolyzing carbapenems using zinc as a cofactor, it is not specifically addressed as a target in the EFSA Opinion, as it is not considered an epidemiological driver of carbapenemase-producing Enterobacterales (CPE) dissemination. In particular, CphA is primarily associated with *Aeromonas* spp. [39], and OXA-58 is a class D (OXA-type) carbapenemase, frequently reported on plasmids and strongly associated with *Acinetobacter* spp. It is mainly relevant in clinical and hospital settings, rather than representing a major driver of CPE dissemination along the food chain [40].

This study presents several limitations. First, in the selected poultry houses, all investigated flocks ended up being raised without antimicrobials; therefore, a true control group was lacking. The only control group available was that of the pilot study. Second, sample and metadata collection during transport and slaughtering were not possible due to restrictions linked to SARS-CoV-2 and avian influenza outbreaks occurring during the study trial. This limitation hindered our ability to assess the relative contribution of these individual stages to the dissemination of AMR on carcasses. Third, although the distance between each poultry house and the slaughtering facility is reported, any event or condition occurring during transport remains unknown. Fourth, we were unable to retrieve information on the use of disinfectants and biocides at the slaughterhouse. The use of these molecules may select for efflux pumps and contribute to multidrug resistance, as well as to co-selection of antibiotic resistance. The impact of different decontamination strategies implemented at the slaughterhouse on poultry meat AMR should be deeply investigated. Fifth, only one slaughterhouse was included both in the present and in the pilot study, which may limit the representativeness of the results. As a follow-up to this study, a further trial will be organized, involving at least three commercial poultry companies. This will enable the inclusion of both conventional and antibiotic-free flocks and provide the opportunity to perform samplings during many transport scenarios and in different slaughterhouses.

Beyond these limitations, the findings of this study indicate that reducing AMR in poultry meat extends beyond farm-level interventions. The benefits of the strategies aimed at reducing AMR at the farm level seem to be compromised during the post-harvest stages. Our results showcase to policy makers that reducing consumer exposure to AMR risk can only be achieved through a true implementation of the One Health Action Plan, which calls for coordinated and multisectoral efforts to mitigate AMR across the entire food systems. For the One Health Action Plan to be effectively implemented and to generate a tangible impact, specific measures should be designed to promote the engagement of poultry companies, willing to share relevant metadata, in research trials.

## Figures and Tables

**Table 1:** Sample groups defined according to the matrix type (source), the poultry house and the sampling period.

Source	Poultry House	Sampling Year	Number of Samples
Caeca	CM	2019	15
Carcasses	CM	2019	15
Caeca	ZR	2019	15
Carcasses	ZR	2019	15
Caeca	CM	2023	22
Carcasses	CM	2023	23
Caeca	ZR	2023	23
Carcasses	ZR	2023	23

**Table 2:** The most represented AMR gene families according to the Comprehensive Antibiotic Resistance Database (CARD). For each gene family, the corresponding antibiotic or antibiotic class to which resistance is conferred and the associated resistance mechanism are reported. Log2FC indicates the magnitude of the difference between caecum and carcass samples. *p*-values were calculated using the Wilcoxon rank-sum test and adjusted for multiple comparisons using the Benjamini–Hochberg false discovery rate (FDR) correction.

ARO Term *	AMR Gene Family	Drug Resistance	Resistance Mechanism	log2FC	*p*-Value	FDR
ARO:3005050	Intrinsic peptide antibiotic-resistant Lps	peptide antibiotics	reduced permeability to antibiotic	−10.446	2.7528 × 10^−22^	4.3433 × 10^−21^
ARO:3005113	AAC(6’)-Ib-cr	multidrug	antibiotic inactivation	−6.692	7.0385 × 10^−21^	9.5187 × 10^−20^
ARO:3007728	OXA-58-like beta-lactamase	penicillin beta-lactam, carbapenem	antibiotic inactivation	−4.603	8.4023 × 10^−11^	6.6285 × 10^−10^
ARO:3007699	OXA-12-like beta-lactamase	penicillin beta-lactam	antibiotic inactivation	−3.906	8.9261 × 10^−8^	4.783 × 10^−7^
ARO:0010004	resistance-nodulation-cell division (RND) antibiotic efflux pump	multidrug	antibiotic efflux	−3.600	1.2933 × 10^−22^	2.1606 × 10^−21^
ARO:3000581	CphA beta-lactamase	Carbapenem	antibiotic inactivation	−3.458	1.4859 × 10^−7^	7.4035 × 10^−7^
ARO:3004251	antibiotic-resistant cya adenylate cyclase	phosphonic acid	antibiotic target alteration	−3.035	4.2109 × 10^−13^	3.8578 × 10^−12^
ARO:3004232	ampC-type beta-lactamase	Cephalosporin, penicillin beta-lactam	antibiotic inactivation	−2.832	2.4497 × 10^−6^	1.0871 × 10^−5^
ARO:0010002	major facilitator superfamily (MFS) antibiotic efflux pump	multidrug	antibiotic efflux	−2.696	3.7094 × 10^−8^	2.1069 × 10^−7^
ARO:0010003	small multidrug resistance (SMR) antibiotic efflux pump	multidrug	antibiotic efflux	−2.660	3.8264 × 10^−10^	2.7864 × 10^−9^
ARO:3000869	streptothricin acetyltransferase (SAT)	nucleoside	antibiotic inactivation	6.742	1.8094 × 10^−25^	4.6715 × 10^−24^
ARO:3007406	APH(3’)	aminoglycoside	antibiotic inactivation	7.499	1.5814 × 10^−24^	3.4547 × 10^−23^
ARO:3004192	CepA beta-lactamase	Cephalosporin	antibiotic inactivation	7.579	6.814 × 10^−25^	1.6126 × 10^−23^
ARO:3000234	glycopeptide resistance gene cluster, vanR	glycopeptide antibiotics	antibiotic target alteration	8.227	5.5485 × 10^−26^	1.7509 × 10^−24^
ARO:3003419	antibiotic-resistant rpsL	Aminoglycoside	antibiotic target alteration	8.260	1.1155 × 10^−20^	1.44 × 10^−19^
ARO:3000202	Cfr 23S ribosomal RNA methyltransferase	multidrug	antibiotic target alteration	9.004	2.6989 × 10^−26^	1.2775 × 10^−24^
ARO:3007702	OXA-184-like beta-lactamase	penicillin beta-lactam	antibiotic inactivation	9.090	4.1378 × 10^−29^	1.1751 × 10^−26^
ARO:3000128	APH(2’’)	Aminoglycoside	antibiotic inactivation	9.247	3.3272 × 10^−28^	4.7246 × 10^−26^
ARO:3004178	23S rRNA with mutation conferring resistance to pleuromutilin antibiotics	Pleuromutilin	antibiotic target alteration	10.209	1.5485 × 10^−27^	1.4659 × 10^−25^
ARO:3000225	ANT(6)	Aminoglycoside	antibiotic inactivation	10.649	1.1095 × 10^−26^	7.8778 × 10^−25^

* ARO Term indicates the unique identifier assigned to each resistance determinant in the Antibiotic Resistance Ontology (ARO) database [25].

**Table 3:** Antibiotic molecules or classes showing mean abundance values in caecum and carcass samples. Log2FC represents the magnitude of the difference between sample sources; negative and positive values indicate higher abundance in carcasses and caeca, respectively. *p*-values were calculated using the Wilcoxon rank-sum test and adjusted for multiple comparisons using the Benjamini–Hochberg false discovery rate (FDR) correction.

Antibiotic Resistance	Mean in Caeca	Mean in Carcasses	Log2FC	*p*-Value	FDR
Fluoroquinolone	31,726.88	404,558.81	−4.237	2.68 × 10^−25^	1.50 × 10^−24^
β-Lactam *	42,018.85	259,028.77	−3.012	1.54 × 10^−19^	4.17 × 10^−19^
Glycylcycline	6615.54	46,447.04	−2.978	6.90 × 10^−14^	1.69 × 10^−13^
Phosphonic acid	3373.00	19,188.06	−2.624	7.72 × 10^−14^	1.74 × 10^−13^
Nucleoside	11,050.11	74,030.71	−2.522	2.04 × 10^−21^	6.11 × 10^−21^
Aminocoumarin	2679.47	15,942.39	−2.417	1.11 × 10^−13^	2.32 × 10^−13^
Rifamycin	9764.72	51,240.72	−1.937	2.65 × 10^−13^	5.11 × 10^−13^
Phenicol	40,199.72	69,998.54	−0.585	2.79 × 10^−10^	4.43 × 10^−10^
Aminoglycoside	203,103.67	378,796.66	−0.583	0.000452	0.000581
Nitroimidazole	455.56	1655.72	−0.067	0.000102	0.000138
Nitrofuran	371.62	1699.54	0.345	0.152811	0.15869
Sulfonamide	1236.78	4706.24	0.382	0.001586	0.001946
Tetracycline	93,967.92	82,817.35	0.512	0.014556	0.017088
Isoniazid-like	385.13	1403.94	0.798	0.067584	0.072990
Diaminopyrimidine	813.96	1658.91	1.633	0.382979	0.382979
Macrolide	162,991.57	81,893.09	1.283	2.79 × 10^−25^	1.50 × 10^−24^
Lincosamide	100,520.12	44,646.39	1.471	6.30 × 10^−25^	2.58 × 10^−24^
Elfamycin	14,781.72	10,151.69	1.650	9.83 × 10^−5^	0.000138
Glycopeptide	31,051.17	12,229.97	1.743	7.65 × 10^−25^	2.58 × 10^−24^
Oxazolidinone	29,164.73	10,399.70	1.906	3.46 × 10^−26^	6.16 × 10^−25^
Pyrazine	78.07	88.32	2.629	2.93 × 10^−6^	4.39 × 10^−6^
Fusidane	526.58	685.80	2.722	0.049592	0.055791
Streptogramin	58,309.53	10,274.17	3.498	1.55 × 10^−25^	1.39 × 10^−24^
Pleuromutilin	32,141.03	4427.93	3.648	4.56 × 10^−26^	6.16 × 10^−25^
Mupirocin-like	154.11	60.09	3.965	3.37 × 10^−11^	5.69 × 10^−11^
Bicyclomycin-like	1207.81	0.00	4.064	9.67 × 10^−12^	1.74 × 10^−11^

* β-Lactam refers to the sum of the abundance of cephalosporin, cephamycin, penam, carbapenem, penem and monobactam.

## Data Availability

All shotgun metagenome sequencing data generated and analyzed in this study are available from the corresponding author upon request (valentina.indio2@unibo.it).

## Abbreviations

The following abbreviations are used in this manuscript:

AMR	Antimicrobial Resistance
EU	European Union
EFSA	European Food Safety Authority
DNA	Deoxyribonucleic Acid
RGI	Resistance Gene Identifier
CARD	Comprehensive Antibiotic Resistance Database
ARO	Antibiotic Resistance Ontology
CPM	Counts Per Million
